# Plasma levels of lipopolysaccharide correlate with insulin resistance in HIV patients

**DOI:** 10.1186/s13098-018-0308-7

**Published:** 2018-01-31

**Authors:** Marcelo Nardi Pedro, Daniela Oliveira Magro, Elizabete Urbano Pinaço Pinto da Silva, Dioze Guadagnini, Andrey Santos, Rogerio de Jesus Pedro, Mario José Abdalla Saad

**Affiliations:** Department of Internal Medicine-FCM, University of Campinas-UNICAMP, Campinas, SP Brazil

**Keywords:** HIV, HAART, Insulin resistance, LPS

## Abstract

**Background:**

In HIV patients using HAART insulin resistance is a central pathophysiological condition that can contribute to the development of diabetes and cardiovascular complications. To examine the role of adipocyte hormones and LPS in insulin resistance in HIV patients, we investigated the role of adiponectin, leptin, visfatin and LPS levels in the insulin resistance of HIV-infected patients treated with HAART.

**Methods:**

This study included 67 HIV positive individuals on HAART and ten healthy controls. All participants performed plasma or serum levels of glucose; insulin; lipids, visfatin, leptin, adiponectin, and LPS. The homeostasis model assessment (HOMA-IR), was used to estimate insulin resistance.

**Results:**

The levels of visfatin, leptin and adiponectin were similar between controls and HIV patients. However, circulating levels of LPS were higher in HIV patients on HAART than in controls. There was a positive correlation between LPS and TG (r = 0.49, p = 0.0001), between LPS and TG/HDL (r = 0.50, p = 0.0001), between LPS and insulin (r = 0.52, p = 0.0003), and between LPS and HOMA-IR (r = 0.52, p = 0.0005), in HIV patients.

**Conclusions:**

Our results showed a clear correlation between plasma LPS and markers of insulin resistance, suggesting a relationship between LPS levels and metabolic alterations, particularly affecting lipids and insulin resistance in HIV patients.

## Background

Over the past 20 years, the introduction of antiretroviral therapy has increased the life expectancy of HIV patients, but it also introduced complicating diseases such as cardiovascular events, renal disease, osteoporosis and metabolic alterations [1–3]. Previous data showed that in patients with HIV in use of HAART the most prevalent metabolic alterations were reduced HDL-cholesterol and hypertriglyceridemia [4]. Among the metabolic complications encountered, insulin resistance is a central pathophysiological condition that can contribute to the development of diabetes and cardiovascular events [5–7]. In patients using HAART, the prevalence of insulin resistance ranges from 35 to 65%, which is higher than in a control population [8–10]. However, the mechanisms by which patients using HAART develop insulin resistance are not completely known [11, 12].

It is well known that changes in body fat distribution are a common finding in HIV-infected patients treated with HAART, associated with increased triglycerides and insulin resistance (IR) [13–21]. In the past, adipose tissue was considered only an energy storage organ, but over the last two decades, it has emerged as an endocrine organ secreting multiple hormones, proteins, and metabolites including inflammatory mediators such as tumor necrosis factor alpha (TNF-α) and IL6 [22–26]. Some products produced by adipocytes (e.g., TNF-α, IL6) can induce insulin resistance. Few, as adiponectin can improve insulin action. Leptin shows a clear correlation with adipose mass. Visfatin plays a controversial role in the action of insulin on adipose tissue [27–29]. However, the role of these hormones in insulin resistance in HIV patients is not yet completely known.

In obesity/type 2 diabetes and other forms of insulin resistance, a subclinical inflammation can have a central role in the induction of insulin resistance [23–26]. This state of subclinical inflammation may be a consequence of alterations in the intestinal microbiota [21, 25–27] which may interfere with intestinal permeability, increasing the absorption of lipopolysaccharide (LPS) leading to increased activation of inflammatory pathways [26, 30–33]. Similarly, previous data showed that LPS circulating levels are increased in HIV patients and correlates with measures of innate immune activation [34, 35]. However, the role of LPS in insulin resistance in HIV patients is not completely known. To examine the role of adipocyte hormones and LPS in reduced insulin sensitivity in HIV patients, in the present study, we investigated the role of adiponectin, leptin, visfatin and LPS plasma levels in the insulin resistance of HIV-infected patients treated with HAART.

## Methods

This was a cross-sectional study developed at the University State of Campinas, from January to July 2007.

### Subjects

A total of 67 HIV positive individuals (47 men and 20 women; age 40.2 ± 9 years old) on HAART and 10 healthy controls adult (6 men and 4 women; age 34 ± 12.1 years old) were recruited at the Department of Internal Medicine, HIV/AIDS Clinical Research Unit of the Infectious Disease.

Inclusion criteria were age greater than 18 years, the presence of HIV infection (according to CDC 1993). All the HIV patients were on HAART. The HIV group was divided into two subgroups based on presence or absence of lipodystrophy, confirmed by a doctor or dietitian in the moment of data collected.

Exclusion criteria were subjects younger than 18 years old or older than 65, body mass index (BMI) > 27, steroids treatment, previous liposuction and similar cosmetic surgery, pregnancy, breastfeeding and diabetes type 1 and 2.

### Anthropometric analysis

All participants underwent an anthropometric evaluation that included the nutritional status, BMI-calculated as weight/height^2^ (kg m^2^), skin fold, abdominal and hip circumferences. Waist circumference was measured to the nearest millimeter using anatomical landmarks.

The presence of lipodystrophy was evaluated according to the Multicenter Aids Cohort Study (MACS). In the HIV group, we asked about weight variation and individual perception on their fat distribution, before and after HIV diagnosis. Our focus was on (a) increased fat under the chin (b) increased fat on the back of the neck (c) increased abdominal girth (d) increased chest or breast fat (e) loss of fat in the face (f) loss of fat in the arms (g) loss of fat in the buttocks (h) loss of fat in the legs. The physician validated information reported by the patient after conducting a thorough physical examination. Fat maldistribution was recorded separately for fat losses and accumulations. Lipodystrophy was categorized as the presence of at least one site with moderate or severe fat loss or presence of at least one site with moderate or severe fat accumulation, excluding isolated abdominal fat accumulation. In summary, the lipodystrophy was defined by patient report (standardized questionnaire) of peripheral lipoatrophy (face, arms, buttocks or legs) with or without central fat accumulation (abdomen, dorsal-cervical fat pad). The diagnosis of central adiposity was defined by a waist-hip ratio (WHR) of > 0.90 in men and > 0.80 in women.

### Laboratory analysis

Plasma and serum were obtained after an overnight fast (12 h), aliquoted and stored at – 80 °C. Plasma glucose levels; hemoglobin A1c; triglycerides; total cholesterol and fractions (LDL-chol; HDL-chol)((Labtest, Brazil) were measured as previously described [36]. Serum was also used to evaluate levels of Visfatin and Adiponectin [AdipoGen Inc, Switzerland], insulin, leptin, and adiponectin [Millipore, USA]) and lipopolysaccharides (LPS [Lonza Inc., USA]). Related to insulin determination, the lowest level of insulin that can be detected by this assay is 1 µU/mL, and the specificity of the analytical test for human insulin is 100% (ED (50) = 0.68 nM). The intra-assay variation is 5.96 ± 1.17 and the inter-assay variation is 10.3 ± 0.9 (EMD Millipore, USA cat#EZHI-14K). The homeostasis model assessment (HOMA-IR) from the formula [glucose (mmol/L) × Insulin (μU/mL)/22.5], was used to estimate insulin resistance.

### Immunological data

The data referred to the immunological HIV group were obtained from medical records. Viral load (copies/ml) and T CD4+ lymphocytes number (cells/mm^3^) were compiled 3 months before or after the beginning of the study, as well as the antiretroviral therapy used for them.

### Statistical analysis

The results were expressed as mean ± standard deviation (mean ± SD). Mann–Whitney test was used to compare the results between two groups, and for more than two groups, we used ANOVA. The Spearman correlation tests were used to check the linear relation between two variables. Statistical significance was assumed if p < 0.05 for all statistical tests. Statistical analyses were used according to SSPS v.16.0 software.

## Results

The anthropometric and clinical characteristics of patients and controls are shown in Table 1. The mean age for HIV patients (40.4 ± 9.4 × 34.0 ± 12.1 years) was slightly but not significantly higher compared to controls. Body mass index (BMI) and waist to hip ratio were similar in controls and HIV patients.

**Table 1:** Characteristic of study population

Variable	Control	HIV
Number	10	67
Male sex (%)	60	59.7
Age (years)	34 ± 12.1	40.4 ± 9.0
BMI	24.3 ± 2.8	22.9 ± 2.7
Waist circumference (cm)	80.1 ± 10.3	82.5 ± 7.0
Total fat (mm)	109.3 ± 39.1	89.5 ± 35.9
Central fat (mm)	78.0 ± 28.0	65.3 ± 25.6
waist/hip	0.8 ± 0.10	0.89 ± 0.07

**Fig. 1:**
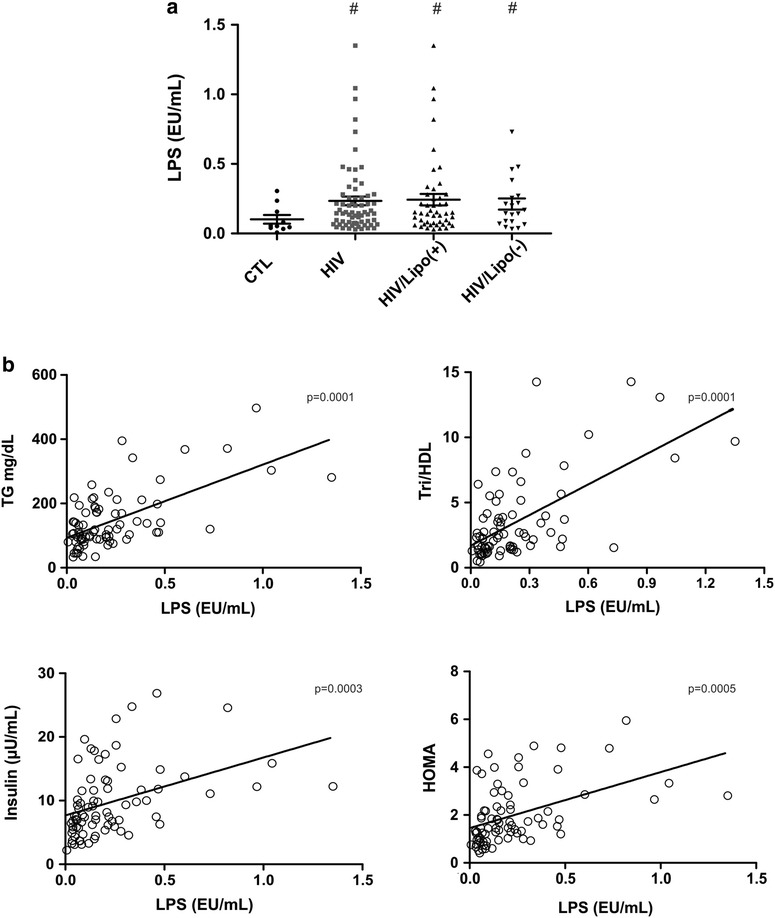
Circulating levels of LPS and Spearman correlation between circulating LPS leaves and selected insulin resistance index. **a** LPS circulating levels in controls (CTL), HIV patients, HIV patients with lipodystrophy (HIV/Lipo(+)) and without lipodystrophy (HIV/Lipo(−)) ^#^p < 0.05 vs. control. **b** Correlation between LPS and TG (Triglycerides) (*r* = 0.49, *p* = 0.0001), between LPS and TG/HDL (*r* = 0.50, *p* = 0.0001), between LPS and insulin (*r* = 0.52, *p* = 0.0003), and between LPS and HOMA-IR (*r* = 0.52, *p* = 0.0005), in the entire group. O: HIV patients

In Table 2 we can observe that fasting plasma glucose in the HIV group (82 ± 7 mg/dL) was similar to controls (79 ± 11 mg/dL). Fasting serum insulin levels were modestly but significantly elevated in HIV patients (10.1 ± 5.4 × 7.0 ± 2.5 μU/mL p > 0.05) and in accordance, the HOMA index was higher in HIV patients (1.9 ± 0.9 × 1.5 ± 0.6 p < 0.05). Fasting plasma triglycerides were higher in the HIV group than in the controls (158 ± 91 × 78 ± 27 mg/dL p < 0.001). Fasting total and LDL cholesterol levels were similar in the HIV and controls. Mean HDL-C was lower in the HIV group than in the control group (50.9 ± 15.7 × 57.2 ± 12.3 mg/dL p < 0.05), and the ratio of triglycerides to HDL was also higher in HIV patients (3.8 ± 3.2 × 1.5 ± 0.7 p < 0.001). All the HIV patients were on stable HAART and plasma HIV-1 viral load was undetectable in 79% of the patients, and the T CD4+ lymphocytes number was over 350 in 95% of these patients. The levels of visfatin, leptin and adiponectin were similar between controls and HIV patients (Table 2). However, circulating levels of LPS were higher in HIV patients than in controls (0.23 ± 0.25 × 0.10 ± 0.1 EU/mL p = 0.01) (Table 2 and Fig. 1).

**Table 2:** Clinical and laboratory of study population

Variables	Control	HIV
Number	10	67
Fasting plasma glucose (mg/dL)	79.2 ± 11	82.2 ± 7
Triglycerides (mg/dL)	78.8 ± 27.5	158.3 ± 91.2**
Total cholesterol (mg/dL)	173.1 ± 19.8	189.0 ± 45
LDL (mg/dL)	101.4 ± 28.6	107.9 ± 36.08
HDL (mg/dL)	57.2 ± 12.3	50.9 ± 15.7*
Visfatin (ng/mL)	4.5 ± 1.2	6.9 ± 6.2
Leptin (ng/mL)	8.7 ± 6.7	6.8 ± 7.5
Adiponectin (μg/mL)	9.1 ± 3.1	9.0 ± 9.14
Trig/HDL	1.5 ± 0.7	3.76 ± 3.18**
Insulin (μU/mL)	7.0 ± 2.5	10.1 ± 5.4*
HOMA	1.5 ± 0.6	1.9 ± 0.9*
LPS (EU/mL)	0.08 ± 0.05	0.23 ± 0.25*

* p < 0.05, ** p < 0.001

In this regard, when we divided the HIV patients into two groups (with lipodystrophy and without lipodystrophy) the increase in LPS circulating levels were more evident in patients with lipodystrophy (0.42 ± 0.30 × 0.10 ± 0.1 EU/mL p = 0.01) than in patients without this abnormality 0.21 ± 0.16 × 0.10 ± 0.1 EU/mL p = 0.03), compared to controls in both situations. The levels of adiponectin were significantly lower in HIV patients with lipodystrophy (7.1 µg/mL × 10.1 µg/mL p < 0.02), but no differences in the levels of leptin or visfatin were observed between the groups.

## Correlations

Related to the index of insulin resistance in HIV patients there was a positive correlation between insulin and TG/HDL (*r* = 0.64, *p* = 0.0001), between HOMA and TG/HDL (*r* = 0.61, *p* = 0.0001) and a negative correlation was seen between adiponectin and TG/HDL (*r* = − 0.50, *p* = 0.0011).

There was a weak inverse correlation between the number of CD4+ T cells and TG/HDL (r = 0.22 p < 0.05), and HOMA (r = 0.23 p < 0.04). However, no correlation was observed between CD4+ and LPS, but in very high LPS levels (> 0.5 EU/ml) there was a clear positive correlation between these two parameters (r = 0.94 p < 0.01).

There was a negative correlation between LPS and HDL (*r* = − 0.49, *p* = 0.0001), and a positive correlation between LPS and TG (*r* = 0.49, *p* = 0.0001), between LPS and TG/HDL (*r* = 0.50, *p* = 0.0001), between LPS and insulin (*r* = 0.52, *p* = 0.0003) and between LPS and HOMA-IR (*r* = 0.52, *p* = 0.0005), in HIV patients on HAART (Fig. 1). We found no significant correlations between BMI and total central fat or LPS levels, between LPS and adiponectin, visfatin or leptin.

## Discussion

In the present study, we investigated the role of hormones produced by adipose tissue and LPS plasma levels, which is a marker of intestinal permeability, in the insulin resistance in HIV patients. Our results demonstrated an increase in LPS levels in HIV patients, suggesting a relationship between LPS levels and metabolic alterations, particularly affecting lipids and insulin resistance in these patients. On the other side, the hormones visfatin, adiponectin, and leptin did not show significant alterations in HIV patients on HAART.

Although glucose clamp is the standard gold method to measure insulin resistance, different studies have shown a close correlation between this method and HOMA for this purpose, and our data showed that HIV patients had higher levels of HOMA. Moreover, the triglyceride to high-density lipoprotein cholesterol (TG/HDL-C) concentration ratio has been shown to be a reliable index of insulin resistance, similar to the fasting serum insulin [37–40]. The TG/HDL-C ratio as a direct measure of insulin resistance in overweight or obese adults was first reported by Reaven et al. and then confirmed in a larger sample [40]. Our data showed that in HIV patients treated with HAART the TG/HDL-C ratio presented a strong correlation with measurements of insulin resistance as fasting insulin and HOMA, and a negative correlation with adiponectin, indicating that in these patients this index can be used as a measurement of reduced insulin sensitivity.

As pointed recently [41], HOMA-IR does not provide a very precise estimate of peripheral insulin action and although not completely proved it might instead measure the ability of insulin to inhibit hepatic glucose production in the fasting state. In this regard, it is interesting that previous data showed that in HIV patients there is an infection of Kupffer cells in the liver which enhanced LPS cell-surface receptors (TLR4) and increased IL-6 and TNF-a expression [42]. Taken together these results with our data showing an increase in LPS levels we can suggest that these alterations can induce a clear hepatic insulin resistance.

The previous review showed that the highest prevalence of lipodystrophy is observed in women [43], associated with lower levels of HDL-cholesterol and insulin resistance. In this regard, estrogens and estrogens receptors have critical roles in adipose differentiation and fat distribution, contributing to explain this difference. However, in our study, the number of women is smaller making a sub-analysis according to gender more difficult.

The unique aspect of our results is the higher levels of plasma LPS in HIV patients treated with HAART and the strong positive correlation between plasma LPS and measurements of insulin resistance such as fasting insulin, HOMA and the TG/HDL-C ratio. It is important to mention that plasma LPS levels were higher in HIV patients treated with HAART with and without lipodystrophy suggesting that circulating LPS may have a role in the reduced insulin sensitivity in these patients, probably in a manner independent of fat storage.

Notably, the gastrointestinal tract is the principal source of lipopolysaccharide (LPS), because of its massive bacterial load compared to other anatomical sites [44]. Also, there is a direct and strong association between plasma LPS levels and the degree of intestinal permeability, in different pathological conditions [33], including patients with HIV. This translocation of LPS into the plasma leads to activation of both innate and adaptive immune responses [34, 35]. The innate immune response initiates through the binding of LPS to Toll-like receptor 4(TLR4), which will induce the release of inflammatory cytokines, resulting in systemic inflammation [45, 46] and insulin resistance, similar to diet-induced obesity in animals and humans [33]. In mice, the subcutaneous infusion of LPS, to reach levels that simulate the translocation of gut bacterial products, has been shown to induce glucose intolerance, higher insulin levels, insulin resistance, and very interesting increase in body weight accompanied by increases in adipose tissue [47, 48]. Whether this increase in circulating levels of LPS in HIV patients on HAART is related to changes in intestinal permeability and changes in intestinal microbiota deserves further investigation.

It is important to mention that although low levels of CD4+ T cells in gut-associated lymphoid tissues may contribute to explain microbial translocation, no inverse correlation between LPS and CD4+ T cells were identified in our study. This may be a consequence of a possible activation of CD4+ cells induced by LPS [42], which may overshadow this negative correlation. In agreement with the increase in CD4+ T cell number induced by LPS, in HIV patients on HARRT with LPS levels higher than 0.5 EU/mL, there was a positive correlation between these parameters.

As previously demonstrated HIV patients on HAART presented dyslipidemia characterized by high triglyceride levels and reduced HDL-cholesterol levels [49]. This result is by previous data that showed that in patients with HIV in use of HAART the most prevalent metabolic alterations were reduced HDL-cholesterol and hypertriglyceridemia [4]. The association of dyslipidemia with high levels of LPS may contribute to increasing the cardiovascular risk in HIV patients on HAART. Previous data showed that increased levels of LPS are associated with endothelial dysfunction in HIV-infected patients [50] and adverse metabolic outcomes [33, 45, 47]. Also, plasma levels of LPS were also associated with progression of atherosclerosis in HIV patients treated with HAART [49] associated with a negative correlation between large HDL particles measured by NMR spectroscopy and LPS in these patients. This negative association is of concern because greater levels of large HDL particles are associated with a reduced risk of CVD and insulin resistance [51], and lower levels of large HDL are associated with endothelial dysfunction and coronary artery disease [52]. It is important to mention that in the SMART study HDL was associated with a decreased risk of coronary artery disease in HIV-patients [53]. Together, this raises the possibility that an increase in LPS levels during prolonged ART may contribute to the increased CVD risk in part due to adverse effects on HDL particles.

There is some weakness in our study that deserves comments: First we correlate LPS circulating levels with insulin resistance, but we did not use the standard gold method to measure insulin resistance as described above. The euglycemic hyperinsulinemic glucose clamp is a relatively complex method and difficult to perform in the number of patients that we studied. Also, at the time we started this study we did not have a methodology to investigate the microbiota of the patients nor the circulating levels of short chain fatty acids (SCFA), which would have allowed us to have better correlations between the changes in microbiota/intestinal permeability with LPS and SCFA.

In summary, our results showed an increase in LPS levels in HIV patients using HAART, and a correlation between plasma LPS and markers of insulin resistance, suggesting a relationship between LPS levels and metabolic alterations, mainly affecting lipids and insulin resistance in these patients.

## Abbreviations

HIV: human immunodeficiency virus
HAART: highly active antiretroviral therapy
IR: insulin resistance
TNF-α: tumor necrosis factor alpha
IL6: interleukin 6
LPS: lipopolysaccharide
BMI: body mass index
WHR: waist-hip ratio
HOMA: Homeostatic model assessment
T CD4+: T-helper cells (cluster of differentiation 4)
TG: triglycerides
TLR4: Toll-like receptor 4
ART: antiretroviral therapy
CVD: cardiovascular disease
